# Supplementary material for: Unmet Supportive Care Needs after Non-Small Cell Lung Cancer Resection at a Tertiary Hospital in Seoul, South Korea
Source: Healthcare (Basel). 2023 Jul 12;11(14):2012. doi: 10.3390/healthcare11142012 (PMC10378778; doi:10.3390/healthcare11142012)
Supplement: Supplementary file 1 [file healthcare-11-02012-s001.zip › healthcare-2486232-supplementary.pdf]

Supplementary Online Content

Supplementary materials

Supplementary Table S1. Correlates of moderate-to-strong level of unmet need of CaSUN-K in each domain in survivors with shorter duration (< 18months).

Supplementary Table S2. Correlates of moderate-to-strong level of unmet need of CaSUN-K in each domain in survivors with longer duration (≥ 18months).

Supplementary Table S1. Correlates of moderate-to-strong level of unmet need of CaSUN-K in each domain in survivors with shorter duration (< 18months).

	Information		Comprehensive Cancer Care		Existential Survivorship		Quality of Life		Relationship		Financial issues	
	OR (95% CI)	aOR* (95% CI)	OR (95% CI)	aOR* (95% CI)	OR (95% CI)	aOR* (95% CI)	OR (95% CI)	aOR* (95% CI)	OR (95% CI)	aOR* (95% CI)	OR (95% CI)	aOR* (95% CI)
Socio-demographic characteristics												
Age												
≥ 65 years old	1.00		1.00		1.00		1.00		1.00		1.00	
< 65 years old	1.22 (0.84, 1.76)		0.93 (0.65, 1.35)		1.09 (0.74, 1.60)		1.03 (0.71, 1.49)		0.94 (0.64, 1.38)		1.49 (0.98, 2.28)	
Sex												
Female	1.00		1.00		1.00		1.00		1.00		1.00	
Male	0.80 (0.55, 1.16)		1.09 (0.75, 1.57)		0.72 (0.49, 1.05)		0.96 (0.66, 1.39)		0.97 (0.66, 0.72)		0.90 (0.60, 1.36)	
Marital status												
Non-married	1.00		1.00		1.00		1.00		1.00		1.00	
Married	0.69 (0.40, 1.20)		1.29 (0.76, 2.20)		0.61 (0.41, 0.91)		0.80 (0.47, 1.36)		0.88 (0.51, 1.52)		0.76 (0.43, 1.35)	
Education level												
vs. ≥ University	1.00		1.00		1.00		1.00		1.00		1.00	
High school	1.15 (0.74, 1.78)		1.09 (0.70, 1.70)		0.99 (0.63, 1.57)		0.99 (0.64, 1.53)		1.13 (0.71, 1.78)		1.14 (0.70, 1.85)	
≤ Middle school	1.18 (0.75, 1.86)		1.01 (0.64, 1.60)		0.90 (0.56, 1.46)		0.89 (0.57, 1.41)		1.00 (0.62, 1.62)		0.74 (0.44, 1.27)	
Employment status												
Employed	1.00		1.00		1.00		1.00		1.00		1.00	
Unemployed	1.21 (0.84, 1.76)		1.10 (0.75, 1.59)		1.56 (1.06, 2.30)		1.12 (0.77, 1.62)		1.43 (0.97, 2.13)		0.75 (0.50, 1.14)	0.55 (0.31, 0.96)
Household monthly income												
< $3,000	1.00		1.00		1.00		1.00		1.00		1.00	
≥ $3,000	0.87 (0.59, 1.29)		0.98 (0.66, 1.45)		0.91 (0.60, 1.37)	1.66 (1.03, 2.68)	0.98 (0.66, 1.45)		0.74 (0.49, 1.12)		1.09 (0.69, 1.71)	
Disease-related characteristics												
Comorbidity												
No	1.00		1.00		1.00		1.00		1.00		1.00	
Yes	1.33 (0.87, 2.03)		1.57 (1.02, 2.40)		1.28 (0.82, 1.98)		1.72 (1.11, 2.67)	1.72 (1.02, 2.89)	1.20 (0.76, 1.88)		0.74 (0.47, 1.18)	0.55 (0.32, 0.96)
Pathologic staging												
0-I	1.00		1.00		1.00		1.00		1.00		1.00	
II	1.06 (0.63, 1.80)		1.09 (0.64, 1.85)		1.21 (0.69, 2.11)		1.46 (0.87, 2.48)		1.39 (0.81, 2.38)		1.45 (0.82, 2.58)	
III	2.44		2.22		2.12		2.49		1.48		1.82	

	(1.30, 4.59)		(1.18, 4.17)		(1.08, 4.17)		(1.38, 4.49)		(0.83, 2.64)		(1.00, 3.33)	
Psychological characteristics												
Fear of Cancer Recurrence												
Low FCR (< 13)	1.00		1.00		1.00		1.00		1.00		1.00	
High FCR (≥ 13)	5.13 (3.44, 7.67)	4.93 (3.16, 7.71)	4.61 (3.10, 6.86)	4.26 (2.74, 6.63)	7.81 (5.06, 12.04)	6.73 (4.19, 10.83)	5.34 (3.52, 8.09)	4.68 (2.87, 7.61)	6.43 (3.99, 10.37)	5.81 (3.40, 9.91)	4.28 (2.59, 7.06)	3.74 (2.12, 6.60)
EORTC-QoL-C30												
Poor Physical function	2.67 (1.64, 4.34)	1.85 (1.04, 3.29)	2.11 (1.31, 3.41)		3.43 (1.93, 6.09)		2.20 (1.40, 3.46)		1.77 (1.12, 2.78)		1.63 (1.01, 2.63)	
Poor Role function	2.44 (1.67, 3.57)		2.32 (1.59, 3.39)		2.32 (1.56, 3.45)		2.08 (1.43, 3.02)		2.18 (1.48, 3.23)		2.32 (1.52, 3.55)	
Poor Emotional function	3.36 (2.22, 5.09)		3.21 (2.12, 4.88)		3.89 (2.44, 6.20)		2.92 (1.97, 4.33)	1.93 (1.14, 3.25)	3.71 (2.47, 5.57)		3.34 (2.18, 5.14)	
Poor Cognitive function	2.99 (1.89, 4.75)	1.81 (1.05, 3.13)	3.16 (1.97, 5.07)	1.97 (1.13, 3.45)	2.94 (1.76, 4.90)		2.59 (1.68, 3.99)		3.01 (1.95, 4.63)	1.70 (1.02, 2.82)	2.92 (1.86, 4.57)	2.28 (1.34, 3.88)
Poor Social function	2.65 (1.80, 3.91)	1.62 (1.01, 2.60)	3.15 (2.11, 4.69)	2.46 (1.54, 3.93)	4.48 (2.86, 7.01)	3.60 (2.14, 6.04)	2.63 (1.79, 3.84)	2.10 (1.29, 3.42)	3.06 (2.06, 4.55)	2.17 (1.36, 3.45)	2.79 (1.82, 4.27)	1.96 (1.19, 3.21)

Abbreviation: CaSUN-K= Korean version of the Cancer Survivors' Unmet Needs, EORTC-QoL-C30=European organization for research and treatment of cancer quality of life core questionnaire, OR=odds ratio, aOR=adjusted odds ratio, CI=confidence interval. *Data are expressed by multivariable logistic models by stepwise backward selection considering the statistical significance level ($p<0.05$).

Supplementary Table S2. Correlates of moderate-to-strong level of unmet need of CaSUN-K in each domain in survivors with longer duration (≥ 18months).

>=18 month	Information		Comprehensive Cancer Care		Existential Survivorship		Quality of Life		Relationship		Financial issues	
	OR (95% CI)	aOR* (95% CI)	OR (95% CI)	aOR* (95% CI)	OR (95% CI)	aOR* (95% CI)	OR (95% CI)	aOR* (95% CI)	OR (95% CI)	aOR* (95% CI)	OR (95% CI)	aOR* (95% CI)
Socio-demographic characteristics												
Age												
≥ 65 years old	1.00		1.00		1.00		1.00		1.00		1.00	
< 65 years old	0.97 (0.68, 1.39)		1.22 (0.85, 1.75)		1.29 (0.90, 1.84)		1.35 (0.92, 1.98)		1.47 (0.98, 2.20)		1.44 (0.91, 2.29)	1.78 (1.05, 3.01)
Sex												
Female	1.00		1.00		1.00		1.00		1.00		1.00	
Male	0.99 (0.69, 1.42)		1.20 (0.83, 1.72)		0.86 (0.60, 1.24)		0.74 (0.51, 1.09)		1.09 (0.72, 1.64)		1.00 (0.63, 1.59)	
Marital status												
Non-married	1.00		1.00		1.00		1.00		1.00		1.00	
Married	0.94 (0.56, 1.59)		1.22 (0.72, 2.06)		0.73 (0.43, 1.24)		0.70 (0.41, 1.19)		1.29 (0.70, 2.39)		0.61 (0.33, 1.12)	
Education level												
vs. ≥ University	1.00		1.00		1.00		1.00		1.00		1.00	
High school	0.86 (0.56, 1.30)		1.08 (0.71, 1.64)		1.12 (0.74, 1.70)		1.00 (0.64, 1.56)		0.74 (0.46, 1.18)		0.85 (0.50, 1.45)	
≤ Middle school	0.98 (0.62, 1.53)		1.04 (0.66, 1.63)		1.11 (0.71, 1.74)		1.19 (0.74, 1.91)		0.70 (0.42, 1.15)		0.97 (0.55, 1.71)	
Employment status												
Employed	1.00		1.00		1.00		1.00		1.00		1.00	
Unemployed	0.95 (0.66, 1.36)		0.74 (0.52, 1.06)	0.61 (0.41, 0.93)	1.10 (0.77, 1.57)		1.06 (0.73, 1.56)		0.92 (0.62, 1.38)		1.31 (0.82, 2.09)	
Household monthly income												
< $3,000	1.00		1.00		1.00		1.00		1.00		1.00	
≥ $3,000	0.95 (0.65, 1.38)		1.08 (0.74, 1.58)		0.71 (0.48, 1.04)		0.88 (0.59, 1.31)		1.10 (0.72, 1.69)		0.51 (0.32, 0.82)	0.48 (0.28, 0.80)
Disease-related characteristics												
Comorbidity												
No	1.00		1.00		1.00		1.00		1.00		1.00	
Yes	0.94 (0.63, 1.41)		0.94 (0.63, 1.41)		1.01 (0.67, 1.51)		0.92 (0.60, 1.42)		0.92 (0.58, 1.44)		0.86 (0.52, 1.42)	
Pathologic staging												
0-I	1.00		1.00		1.00		1.00		1.00		1.00	
II	1.44 (0.87, 2.38)	1.86 (1.05, 3.29)	1.08 (0.66, 1.79)		1.26 (0.76, 2.09)		1.29 (0.76, 2.17)		1.56 (0.91, 2.65)	1.94 (1.07, 3.52)	1.19 (0.64, 2.23)	
III	0.69		0.78		1.27		1.33		0.80		1.49	

	(0.36, 1.32)		(0.42, 1.47)		(0.68, 2.37)		(0.70, 2.52)		(0.38, 0.45)		(0.72, 3.09)	
Psychological characteristics												
Fear of Cancer Recurrence												
Low FCR (< 13)	1.00		1.00		1.00		1.00		1.00		1.00	
High FCR (≥ 13)	4.17 (2.84, 6.13)	4.03 (2.62, 6.20)	3.97 (2.71, 5.81)	4.00 (2.62, 6.09)	6.51 (4.38, 9.66)	6.17 (3.98, 9.56)	4.58 (2.98, 7.02)	3.90 (2.79, 5.46)	4.52 (2.85, 7.18)	4.19 (2.51, 6.99)	3.42 (2.03, 5.73)	2.48 (1.41, 4.35)
EORTC-QoL-C30												
Poor Physical function	1.73 (1.03, 2.89)		1.55 (0.93, 2.59)		2.62 (1.49, 4.59)		1.83 (1.09, 3.08)		1.64 (0.96, 2.80)		1.65 (0.91, 2.98)	
Poor Role function	1.87 (1.28, 2.74)		1.65 (1.13, 2.41)		1.88 (1.28, 2.77)		2.09 (1.41, 3.09)		2.27 (1.51, 3.42)		2.40 (1.51, 3.81)	
Poor Emotional function	3.40 (2.22, 5.19)	2.05 (1.24, 3.38)	2.82 (2.86, 4.29)	2.01 (1.26, 3.20)	3.84 (2.44, 6.05)	2.09 (1.21, 3.62)	4.08 (2.67, 6.23)	1.86 (1.30, 2.65)	3.41 (2.21, 5.24)	2.48 (1.55, 3.98)	4.00 (2.49, 6.43)	2.78 (1.66, 4.65)
Poor Cognitive function	2.43 (1.61, 3.69)		1.95 (1.29, 2.94)		2.56 (1.66, 3.94)		1.90 (1.25, 2.88)		1.82 (1.18, 2.81)		1.94 (1.19, 3.14)	
Poor Social function	2.62 (1.75, 3.93)		2.40 (1.60, 3.58)		3.61 (2.34, 5.57)	1.96 (1.16, 3.34)	3.83 (2.53, 5.78)	2.00 (1.42, 2.80)	2.78 (1.82, 4.24)		2.12 (1.32, 3.40)	

Abbreviation: CaSUN-K= Korean version of the Cancer Survivors' Unmet Needs, EORTC-QoL-C30=European organization for research and treatment of cancer quality of life core questionnaire, OR=odds ratio, aOR=adjusted odds ratio, CI=confidence interval. *Data are expressed by multivariable logistic models by stepwise backward selection considering the statistical significance level ($p<0.05$).
